# Comparing methods to estimate incremental inpatient costs and length of stay due to methicillin-resistant *Staphylococcus aureus* in Alberta, Canada

**DOI:** 10.1186/s12913-019-4578-z

**Published:** 2019-10-24

**Authors:** Erin Kirwin, Marie Varughese, David Waldner, Kimberley Simmonds, A. Mark Joffe, Stephanie Smith

**Affiliations:** 1 Alberta Ministry of Health, Edmonton, Alberta Canada; 2 Department of Medicine, University of Alberta, Edmonton, Alberta Canada; 3 School of Public Health, University of Alberta, Edmonton, Alberta Canada; 4 Department of Community Health Sciences, University of Calgary, Calgary, Alberta Canada; 5 Alberta Health Services, Edmonton, Alberta Canada

**Keywords:** Methicillin-resistant *Staphylococcus aureus*, Hospital acquired infection, Community acquired infection, Cost of illness, Incremental cost, Semilogarithmic ordinary least squares model, Log linked generalized linear model, Randomized matching

## Abstract

**Background:**

Methicillin-resistant *Staphylococcus aureus* (MRSA) is an opportunistic bacterial organism resistant to first line antibiotics. Acquisition of MRSA is often classified as either healthcare-associated or community-acquired. It has been shown that both healthcare-associated and community-acquired infections contribute to the spread of MRSA within healthcare facilities. The objective of this study was to estimate the incremental inpatient cost and length of stay for individuals colonized or infected with MRSA. Common analytical methods were compared to ensure the quality of the estimate generated. This study was performed at Alberta Ministry of Health (Edmonton, Alberta), with access to clinical MRSA data collected at two Edmonton hospitals, and ministerial administrative data holdings.

**Methods:**

A retrospective cohort study of patients with MRSA was identified using a provincial infection prevention and control database. A coarsened exact matching algorithm, and two regression models (semilogarithmic ordinary least squares model and log linked generalized linear model) were evaluated. A MRSA-free cohort from the same facilities and care units was identified for the matched method; all records were used for the regression models. Records span from January 1, 2011 to December 31, 2015, for individuals 18 or older at discharge.

**Results:**

Of the models evaluated, the generalized linear model was found to perform the best. Based on this model, the incremental inpatient costs associated with hospital-acquired cases were the most costly at $31,686 (14,169 – 60,158) and $47,016 (23,125 – 86,332) for colonization and infection, respectively. Community-acquired MRSA cases also represent a significant burden, with incremental inpatient costs of $7397 (2924 – 13,180) and $14,847 (8445 – 23,207) for colonization and infection, respectively. All costs are adjusted to 2016 Canadian dollars. Incremental length of stay followed a similar pattern, where hospital-acquired infections had the longest incremental stays of 35.2 (16.3–69.5) days and community-acquired colonization had the shortest incremental stays of 3.0 (0.6–6.3) days.

**Conclusions:**

MRSA, and in particular, hospital-acquired MRSA, places a significant but preventable cost burden on the Alberta healthcare system. Estimates of cost and length of stay varied by the method of analysis and source of infection, highlighting the importance of selecting the most appropriate method.

## Background

Methicillin-resistant *Staphylococcus aureus* (MRSA) is an opportunistic bacterial organism resistant to first line antibiotics such as methicillin, oxacillin, penicillin, and first generation cephalosporins [1]. MRSA can asymptomatically colonize skin and mucosal surfaces of healthy people but can also cause infection in many sites including wounds, skin, soft tissue, bones, and joints. Acquisition of MRSA is often classified as healthcare-associated or community-acquired. It has been shown that both healthcare-associated and community-acquired infections contribute to the spread of MRSA within hospital facilities, and that timely screening and hand hygiene strategies are important in controlling transmission [2].

The cost and resource burdens of MRSA are substantial. The total direct healthcare cost attributable to MRSA in Canada has been estimated at 82 million in 2004, and was projected to reach 129 million Canadian dollars by 2010 [3]. In 2012, the rate of MRSA per 10,000 patient days was 2.2, a slight decline from the prior 5 year period [2]. Several studies have shown that MRSA infection in hospital inpatients is associated with increased length of stay (LOS), and correspondingly higher costs prior to discharge [4–19].

Studies estimating healthcare costs associated with MRSA use case cohort or statistical techniques to either (i) compare MRSA cases to Methicillin-susceptible *Staphylococcus aureus* (MSSA) cases, and estimate the cost of microbial resistance [4–9], or (ii) compare MRSA cases to inpatients without MRSA [10–12]. The remaining studies used chart review or additive methods to isolate inpatient costs attributable to MRSA [13–19]. Most of these studies are based upon data that are at least 10 years old (20 years old, in the case of the Canadian studies), and few study MRSA cases that are both community and hospital-acquired.

The aim of this study was to generate a current Canadian estimate of incremental inpatient costs due to community and hospital-acquired MRSA colonization and infection. Two estimation methods were implemented: a matching analysis, and multivariate regression models (selecting between a semilogarithmic ordinary least squares model and a log linked generalized linear model). Nosocomial MRSA transmission is preventable through hand hygiene, environmental cleaning and disinfection, use of personal protective equipment, and contact precaution strategies. The estimates in this study were generated for use in a forthcoming modeling study estimating the cost effectiveness of various strategies to prevent nosocomial MRSA colonization and infection.

## Methods

### Setting

This study was performed at Alberta Ministry of Health (Edmonton, Alberta), with access to administrative data holdings and clinical MRSA data collected at the Royal Alexandra and University of Alberta Hospitals, acute care facilities with 869 and 885 beds, respectively.

### Study design

A retrospective cohort study of patients at the Royal Alexandra and University of Alberta Hospitals with MRSA were identified using the provincial infection prevention and control database, ProvSurv [20], from January 1, 2011 to December 31, 2015. For the matching analyses, cases were compared to matches to estimate the incremental cost of MRSA colonization and infection, while for the multivariate regression models, parameter estimates were used to estimate cost for cases with and without MRSA, and the incremental cost of MRSA infection was calculated as difference between these estimates.

### MRSA screening protocol

MRSA screening was conducted in accordance with local Infection Prevention and Control (IPC) protocols, which mandates screening at admission for patients who have either received hemodialysis, been an inmate at a correctional facility, or have been admitted to a healthcare facility within the preceding 6 months [21]. Screening beyond the time of admission varied and was performed at the discretion of the IPC committee in response to documented in-hospital MRSA transmission and outbreaks. Screening specimens were collected from the nares, groin and when present drainable wounds and subsequently plated on MRSA*Select*™ media, which has been shown to be a reliable method to routinely screen patients for MRSA colonization [22]. Records for positive cases were classified according to a provincial protocol and stored in ProvSurv [23].

### Data collection

ProvSurv has complete capture for all MRSA cases identified in any of the 106 acute care facilities in the province of Alberta. The data extracted from ProvSurv included patient demographic information, infection status (colonized or infected), and sample date. These records were linked to inpatient records from the Discharge Abstract Database at Alberta Health. Individuals under the age of 18 at discharge were excluded, as were all cases for which there was no case costing from the case cohort.

**Fig. 1:**
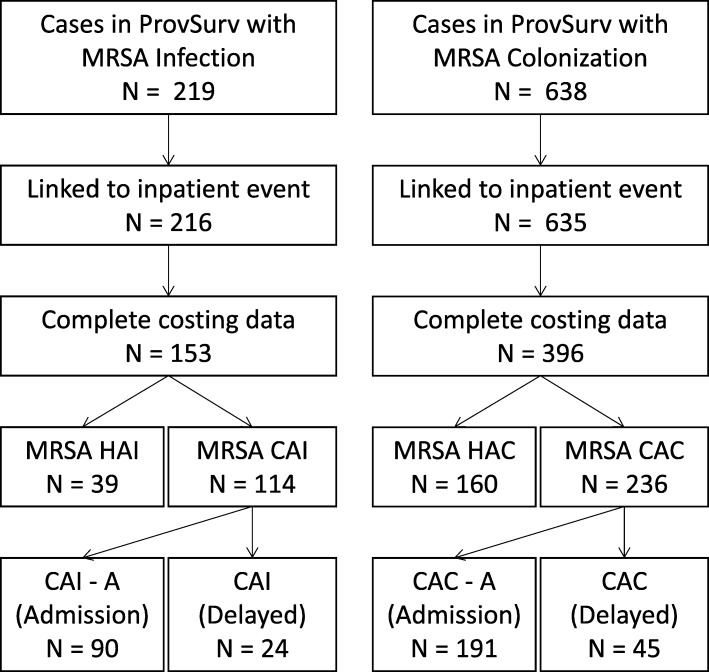
Flowchart depicting case exclusion from study

The cases were split into six case categories: by infection status, and source of infection based on the national case definition [24] used within the ProvSurv database. MRSA cases are referred to as hospital-associated colonization (HAC), hospital-associated infection (HAI), community-acquired colonization (CAC) and community-acquired infection (CAI). Community-acquired cases were split further by those that were identified upon admission (< 24 h following admission), or following admission. Cases identified upon admission were distinguished with an ‘-A’ suffix (ie, CAC-A and CAI-A). Figure 1 depicts case exclusion from the study, and further detail on case classification is given in Additional file 1: Table S1.

A MRSA-free cohort of 577,238 inpatient discharges from the same facilities and care units between January 1, 2011 to December 31, 2015 of patients over 18 years at discharge with complete case costing were extracted and appended to the case dataset.

For the full dataset with both case and matched cohort records, transfers were combined into one event, the number of procedures for each inpatient event were calculated as the total number of procedure codes on the inpatient record, and an individual Charlson-like comorbidity index [25] calculated from a public algorithm [26] was linked based on the year of admission. Inpatient costs were estimated using patient-specific consumption-based costing which is completed at both facilities included in this study [27]. Physician services pertaining to each record were collected from the physician claims dataset and tabulated along with inpatient case costing to calculate the total cost of each inpatient event. All costing data were adjusted to 2016 Canadian dollars using the Alberta Consumer Price Index, according to Canadian Agency for Drugs and Technologies in Health (CADTH) guidelines [28].

### Analytic approach

To determine the difference in healthcare costs and LOS between the cases and the MRSA-free cohort by case category, matching and regression analytic techniques were evaluated. This study was undertaken to generate cost estimates for a forthcoming modeling study evaluating the cost effectiveness of interventions to reduce the spread of MRSA within hospitals. An agent based mathematical model has been developed to simulate the spread of MRSA within hospital units, where inpatients exist in one of the six case categories. This model will be used in a cost effectiveness analysis (CEA) for interventions to prevent nosocomial MRSA infections. The model requires inputs for total cost and LOS by case category. Therefore, the aforementioned case categories were used for the estimation techniques evaluated in this study.

### Matching analysis

A randomized, coarsened exact matching algorithm was applied to the MRSA cases to generate pairs of cases and matches based on five-year age group, sex, urban type (incorporating homeless status), Charlson comorbidity index (binned as 0–2, 3–4, 5–6, 7+), number of procedures (binned as 1, 2, 3, 4, 5–6, 7–9, 10+), and facility. The year of admission was not included in the matching criteria because costs were adjusted for inflation, and both hospitals employed similar infection prevention and control strategies for the duration of the study period. Ten sets of matches were generated and combined to account for variation.

Mean and standard deviation values were calculated for each variable. Difference in means between cases and matches were tested using the nonparametric Wilcoxon rank sum test for independent samples.

### Multivariate regression model analyses

Two regression models were fitted using the MRSA-free cohort dataset with no matching. The models were specified with the total cost and LOS for the inpatient visit, or its natural log, as the dependent variable. Indicators for colonization, infection, hospital-acquired MRSA, and diagnosis on admission, Charlson comorbidity index, number of procedures, age, and sex were specified as independent variables.

The first regression model was a semilogarithmic ordinary least squares model (semilog OLS) with the natural log of the total inpatient cost or LOS as the dependent variable, and independent variables as described above. The second model was a log linked generalized linear model (GLM). The distribution family was selected using a modified Park test [29]. Each model was specified with total inpatient cost and LOS as the dependent variable, with the independent variables as described above.

### Cost and LOS estimation

In the matching approach, mean total costs and LOS of for the MRSA-free cohort were subtracted from the mean values of the cases for each case category to estimate the incremental cost of MRSA colonization and infection.

For the regression models, the method of recycled predictions was used to estimate total cost and LOS [30]. Results were retransformed from log scale [31, 32].

## Results

### Matching analysis

The matching process returned a cohort that was predictably similar in age, sex, homelessness status, Charlson comorbidity index, and number of procedures to the case cohort. The mean and standard deviation for selected characteristics and per diem costs were calculated for colonized and infected cases and their matches (Table 1). Overall, the total cost was significantly higher (*p* < 0.001; Table 1) among hospital-acquired cases compared to matches, and higher at a *p* < 0.05 significance level for community-acquired cases. For all case categories, total cost per diem was not numerically or statistically different between cases and matches, except for HAC and CAI cases where matches cost more than controls, significant at a *p* < 0.001 and a *p* < 0.01 level, respectively.

**Table 1:** Mean and Standard Deviation of Selected Characteristics and per Diem Costs for Cases and Matches

			Female (N)	LOS (days)	Full Cost ($)	Full Cost per Diem ($)	Age (Years)
HAC	CASE	152	50	47.07 (49.9)	87,169.36 (124,352.98)	2045.00 (1573.69)	68.09 (18.1)
	MATCH	1510	490	14.19 (26.1)	29,622.79 (52,720.39)	2846.78 (2853.81)	67.82 (18.2)
	*p* value			<.0001	<.0001	<.0001	0.7677
HAI	CASE	37	16	56.08 (59.8)	147,457.83 (136,521.87)	3094.71 (2138.34)	55.70 (20.5)
	MATCH	370	160	27.53 (41.2)	81,255.88 (154,957.10)	2923.51 (1852.18)	55.31 (20.4)
	*p* value			<.0001	<.0001	0.9212	0.9679
CAC	CASE	44	20	11.07 (15.0)	30,257.82 (57,664.19)	2260.04 (1471.44)	53.95 (19.7)
	MATCH	440	200	11.52 (27.8)	25,315.65 (68,942.20)	2160.62 (1620.05)	53.98 (19.2)
	*p* value			0.0079	0.0134	0.5974	0.9734
CAI	CASE	23	12	24.17 (24.5)	66,334.35 (84,819.14)	2234.48 (1051.56)	53.91 (17.9)
	MATCH	230	120	10.44 (20.4)	27,859.56 (39,070.72)	3716.91 (3196.77)	53.70 (16.6)
	*p* value			<.0001	0.0274	0.0092	0.9988
CAC-A	CASE	187	72	11.36 (21.4)	26,516.53 (43,379.84)	2842.90 (2447.30)	50.72 (19.1)
	MATCH	1860	710	9.22 (21.2)	17,748.70 (37,594.47)	2601.54 (2327.25)	50.78 (18.7)
	*p* value			<.0001	0.0001	0.069	0.8602
CAI-A	CASE	84	28	16.75 (22.6)	36,933.50 (56,716.14)	2556.93 (2311.21)	50.55 (16.4)
	MATCH	840	280	9.29 (18.3)	17,282.27 (24,006.20)	2553.57 (2172.21)	50.43 (16.6)
	*p* value			<.0001	<.0001	0.8968	0.9131

*HAC* Hospital-Acquired Colonization, *HAI* Hospital-Acquired Infection, *CAC* Community-Acquired Colonization, *CAI* Community-Acquired Infection, *CAC-A* Community-Acquired Colonization Identified upon Admission, *CAI-A* Community-Acquired Colonization Identified upon Admission

**Table 2:** Generalized Linear Model (GLM) Regression Results for Cost and LOS

	Cost		LOS	
	Coefficient (St. Err.)	*p* value	Coefficient (St. Err.)	*p* value
Intercept	8.4427 (0.0041)	<.0001	0.8570 (0.0047)	<.0001
Colonized	0.3888 (0.1083)	0.0003	0.3391 (0.1312)	0.0097
Infected	0.6698 (0.1157)	<.0001	0.7177 (0.1403)	<.0001
Hospital-Acquired	0.7216 (0.1214)	<.0001	1.0209 (0.1472)	<.0001
Admission	0.0684 (0.1156)	0.5539	0.0752 (0.1398)	0.5906
Charlson	0.0567 (0.0005)	<.0001	0.0807 (0.0006)	<.0001
Age	0.0121 (0.0001)	<.0001	0.0151 (0.0001)	<.0001
Gender	−0.2050 (0.0025)	<.0001	−0.2214 (0.0031)	<.0001
Procedure Count	0.1967 (0.0006)	<.0001	0.0762 (0.0006)	<.0001

### Multivariate regression models

The modified Park test indicated that Gamma was the best distribution family for the GLM. Following the hypothesis testing algorithm recommended by Manning and Mullahy [29], tests indicated that the GLM was preferred to the semilog OLS model. Full details on the results from these tests are available in Additional file 1: Table S2. Coefficient estimates from the GLM for cost and LOS are given in Table 2. The estimated incremental cost for HAI ($47,016) was highest overall, compared to HAC ($31,686), CAC ($7397), CAI ($14,847), CAC-A ($9023) and CAI-A ($17,001).

**Table 3:** Estimated Case and Incremental Cost by Analytic Method, 2016 dollars (95% CI)

	GLM Regression		Matched	
	Case	Incr.	Case	Incr.
HAC	47,251 (29322–76,148)	31,686 (14169–60,158)	87,169 (67241–107,098)	57,547 (40279–74,814)
HAI	62,581 (38278–102,322)	47,016 (23125–86,332)	147,458 (101939–192,976)	66,202 (36524–95,880)
CAC	22,962 (18077–29,170)	7397 (2924–13,180)	30,258 (12726–47,789)	4942 (− 6130–16,014)
CAI	30,412 (23598–39,197)	14,847 (8445–23,207)	66,334 (29656–103,013)	38,475 (6872–70,077)
CAC-A	24,588 (15433–39,176)	9023 (280–23,186)	26,517 (20258–32,775)	8768 (4219–13,316)
CAI-A	32,566 (20147–52,642)	17,001 (4994–36,652)	36,934 (24625–49,242)	19,651 (8969–30,334)

### Comparing matching and regression results

Table 3 describes the case and incremental costs estimated by the GLM and the matched methods. The GLM predicted lower incremental costs than the matching method, except for the CAC and CAC-A cases. Table 4 presents similar results for LOS.

**Table 4:** Estimated Case and Incremental LOS by Analytic Method, days (95% CI)

	GLM Regression		Matched	
	Case	Incr.	Case	Incr.
HAC	29.21 (16.44–51.91)	21.71 (9.16–44.19)	47.07 (39.07–55.07)	32.88 (26.2–39.56)
HAI	42.65 (23.58–77.16)	35.16 (16.3–69.44)	56.08 (36.16–76.01)	28.55 (12.84–44.27)
CAC	10.52 (7.9–14.02)	3.03 (0.62–6.29)	11.07 (6.49–15.64)	−0.45 (−2.42–1.52)
CAI	15.37 (11.33–20.83)	7.87 (4.05–13.11)	24.17 (13.58–34.77)	13.73 (5.79–21.68)
CAC-A	11.35 (6.48–19.88)	3.85 (−0.8–12.15)	11.36 (8.27–14.45)	2.13 (0.01–4.26)
CAI-A	16.57 (9.29–29.54)	9.07 (2.01–21.82)	16.75 (11.84–21.66)	7.46 (3.79–11.13)

The results from the GLM indicate that hospital-acquired cases are the most expensive to manage (incremental cost of $31,686 and $47,016 CAD for colonization and infection, respectively), and infection is more expensive to manage than colonization. Similarly, hospital-acquired cases had the longest incremental stays of 21.7 and 35.2 days for colonization and infection, respectively. The results from the matching analysis corroborated these findings, but the incremental cost estimates for all case cohorts except for HAC and HAI were at least twice as high as most of the regression model estimates.

## Discussion

Analytic methods were used to estimate the incremental cost for healthcare-associated and community-acquired colonization and infection in Alberta between 2011 and 2015. There are many challenges to estimating the cost of health care-associated infections, [33, 34] and estimating the incremental cost of MRSA by infection type is no exception. Time-dependent bias, present in both estimation techniques, will likely overstate the incremental cost and LOS related to MRSA colonization and infection in our results.

### Selecting the superior estimation method

The GLM method is superior to the matching method because (i) including additional covariates in regression models will not result in excluding cases from the study, (ii) regression methods allow for the inclusion of Charlson index and number of procedures as continuous variables, and (iii) by adjusting regression estimates using recycled predictions, the impact of individual factors making cases more likely to contract MRSA are included in final estimates. These points are discussed in greater detail below.

Matching methods rely on the assumption that by matching on key variables, the remaining difference in cost can be attributed to infection or disease. This method suffers from two biases: i) not all factors contributing to increased costs for the case cohort can be captured by the matching variables, and ii) attempts to increase control by adding matching variables will result in cases being selected out of the study [33, 34]. Additional matching variables were evaluated for inclusion, but these lead to significant case loss, and it was rarely possible to match cases to more than one individual. Regression methods are better suited to deal with this bias as they control for these factors at the analysis stage rather than at the design stage [33, 34].

The implementation of explanatory variables differed between the matched and regression methods. The Charlson-like index was selected for inclusion as it does not include any categories related to hospital infections, therefore providing an accurate indication of health status around the time of the hospital stay excluding the effect of the infection, and because it can be interpreted on a continuous scale. It is preferable to other health status indices such as the Clinical Risk Grouper or the Case Mix Grouper, both of which would have to be input as a categorical variable in the regression models, and could capture hospital-acquired infections. In the matched method, cases were matched on binned Charlson index, while the more flexible nature of the regression models allowed for the Charlson index to be implemented as a continuous variable.

The number of procedures was a limitation for the matched method. Similar to the Charlson index, they were implemented as a binned categorical variable. By matching on total procedures, both prior to and following MRSA diagnosis for cases, some procedures related to the treatment of MRSA could be included in the matching criteria. This would result in matches with a disproportionately higher number of procedures being matched, likely increasing the estimated cost of the hospital stay for matches, and understating the incremental cost of MRSA colonization and infection. This is not an issue in the regression models, where the continuous nature of the variable allows the effect of MRSA status and number of procedures to be separated.

Further, the method of recycled predictions estimates incremental cost for all individuals in the case cohort, removing some of the bias associated with those most likely to become infected or colonized with MRSA [30].

The results of the GLM regression are referenced for the remainder of the discussion.

### Cost estimate comparisons to other studies

While the results from the GLM model are within the expected range of values from the literature [4–19], this study is unique in that it provides estimates for six MRSA case categories, estimates incremental costs of MRSA rather than methicillin resistance, and is one of a few to contribute a concrete example on the impact of different estimation methods. Several studies estimate the cost of MRSA; however, many compare MRSA cases to Methicillin-susceptible *Staphylococcus aureus* (MSSA) cases, rather than a MRSA-free cohort [4–9], making them inappropriate for inputs in our forthcoming CEA. The choice and inclusion of explanatory variables related to both LOS and cost varied by study. Several studies used propensity score matching, which may introduce unnecessary bias in final estimates [35, 36]. Many of the studies were undertaken as stand-alone analyses, whereas our study was designed to generate outputs in a forthcoming modeling study.

Additional file 1 Table S3 presents results of other studies estimating the attributable or total cost of MRSA colonization and infection, using a variety of estimation techniques. We built upon an existing table [37], adding more recent English language literature. The Alberta CPI index [38], and historical exchange rates were used to adjust costs to 2016 Canadian dollars. If available, attributable or incremental costs of MRSA are reported. Otherwise, estimates for the total cost of MRSA patients are given.

Estimates from this study are in line with other Canadian studies. Kim et al. [14] reported an attributable cost of infection between our estimates for HAI and CAI. Their cost estimate for colonized cases is much lower than any of our estimates. Papia et al. [13] estimated a cost for colonized cases diagnosed on admission or later very close to our estimate for CAC. Converted European estimates for costs attributable to MRSA infection range from $5150 [17] to $18,740 [19], and are close to our estimate for CAI.

Estimates from American studies were generally higher than ours, which is expected as it has been demonstrated that equivalent services cost approximately 1.4 times more in the USA than in Canada [39]. Estimates for the total cost of hospitalization with MRSA on admission in Reed et al. [9] and Ben-David et al. [5] are higher than our comparable figures for CAI-A and HAI, but adjusting for expected cost differences between the two countries, the estimate from Reed et al. (converted $41,400) falls just below our HAI cost estimate, while the estimate from Ben-David et al. (converted $77,700) is much higher than our estimate for the incremental cost of HAIs.

In another study from the USA, Nelson et al. [10] controlled for time-dependent bias by using data that have costs tabulated at discrete time intervals. Their estimate for the incremental cost of nosocomial MRSA infections ($28,860) is lower than ours. When adjusting for price level, this estimate becomes $20,610. Nelson et al. estimated that time-dependent bias increases costs by 31.5%. Applying this to our estimate for HAI ($31,690) becomes $21,700, close to the estimate in [10] adjusted for health system price level.

### Study limitations

MSSA and other hospital-acquired and multidrug resistant infections were not actively excluded from our MRSA-free cohort. This could result in underestimating incremental costs if a MSSA case was included in the MRSA-free cohort. However, most studies estimated the costs of MSSA infection to be significantly lower than MRSA infection.

Data sources for this study are not available on a daily basis, so methods to address time-dependent bias, like those in [10] were not implementable in this study. A new data source that will contain daily cost estimates is expected to be produced for the province of Alberta. Future publication using daily cost estimates will allow for comparison to the methods in this study, and a more precise estimate for the incremental cost of MRSA. In the meantime, it may be advisable to reduce the incremental cost estimates found here by the factor calculated in [10] of 31.5%.

## Conclusion

MRSA, and in particular, hospital-acquired MRSA, places a significant but preventable cost burden on the Alberta healthcare system. The estimates from this analysis provide a needed update to Canadian estimates. Comparing the results from the matched and regression analyses, a GLM provided the best estimates for incremental hospital costs in MRSA cases, and this model performed well compared to other studies in reducing time-dependency bias. Differences in incremental cost estimates by MRSA case category highlight the importance of studying these groups separately. The results of this study will be useful in future work to evaluate the cost effectiveness of infection prevention and control activities.

## Supplementary information

**Additional file 1: Table S1.** Case Category Names and Database Criteria. **Table S2.** Application of Model Comparison Algorithm in Manning and Mullahy 2001 [29]. **Table S3.** Cost per MRSA Case, updated from Tübbicke et al. 2012 [36].

## Data Availability

The datasets generated during and/or analysed during the current study are not publicly available due to provincial legislation but are available from the corresponding author on reasonable request, subject to the Alberta Health Information Act.

## Abbreviations

CAC: Community-acquired colonization
CAC-A: Community-acquired colonization identified on admission
CADTH: Canadian Agency for Drugs and Technologies in Health
CAI: Community-acquired infection
CAI-A: Community-acquired infection identified on admission
CEA: Cost Effectiveness Analysis
GLM: Log linked generalized linear model
HAC: Healthcare associated colonization
HAI: Healthcare associated infection
LOS: Length of stay
MRSA: Methicillin-resistant *Staphylococcus aureus*
MSSA: Methicillin-susceptible *Staphylococcus aureus* (MSSA)
semilog OLS: Semilogarithmic ordinary least squares model
