# Correction to: Comparing methods to estimate incremental inpatient costs and length of stay due to methicillin-resistant *Staphylococcus aureus* in Alberta, Canada

**DOI:** 10.1186/s12913-019-4877-4

**Published:** 2019-12-31

**Authors:** Erin Kirwin, Marie Varughese, David Waldner, Kimberley Simmonds, A. Mark Joffe, Stephanie Smith

**Affiliations:** 1 Alberta Ministry of Health, Edmonton, Alberta Canada; 2 Department of Medicine, University of Alberta, Edmonton, Alberta Canada; 3 School of Public Health, University of Alberta, Edmonton, Alberta Canada; 4 Department of Community Health Sciences, University of Calgary, Calgary, Alberta Canada; 5 Alberta Health Services, Edmonton, Alberta Canada

**Correction to: BMC Health Serv Res (2019) 19:743**

**https://doi.org/10.1186/s12913-019-4578-z**

In the original publication of this article [1], the authors want to add the following sentence in the Acknowledgement section:

**The authors thank the Infection Prevention and Control (IPC) Program at Alberta Health Services for providing access to the IPC surveillance platform data.**
