# Supplementary material for: Comparing methods to estimate incremental inpatient costs and length of stay due to methicillin-resistant Staphylococcus aureus in Alberta, Canada
Source: BMC Health Serv Res. 2019 Oct 24;19:743. doi: 10.1186/s12913-019-4578-z (PMC6813095; doi:10.1186/s12913-019-4578-z)
Supplement: Supplementary file 1 — Additional file 1: Table S1. Case Category Names and Database Criteria. Table S2. Application of Model Comparison Algorithm in Manning and Mullahy 2001 [29]. Table S3. Cost per MRSA Case, updated from Tübbicke et al. 2012 [36]. [file 12913_2019_4578_MOESM1_ESM.docx]

Additional File 1.

**Table A1.** Case Category Names and Database Criteria

| **Full name** | **Acronym** | **Provsurv Database Criteria** |
| --- | --- | --- |
| Hospital Acquired Colonization | HAC | Severity: 'Colonized' or 'Unknown'  Classification: 'Hospital acquired' |
| Hospital Acquired Infection | HAI | Severity: 'Infection'  Classification: 'Hospital acquired'. |
| Community Acquired Colonization | CAC | Severity: 'Colonized' or 'Unknown'  Classification: Not 'Hospital or 'Healthcare acquired'.  The interval between admission date and screening date must be greater than or equal to one. |
| Community Acquired Infection | CAI | Severity: 'Infection'  Classification: Not 'Hospital or 'Healthcare acquired'  The interval between admission date and screening date must be greater than or equal to one. |
| Community Acquired Colonization – Admission | CAC-A | Severity: 'Colonized' or 'Unknown'  Classification: Not 'Hospital or 'Healthcare acquired'  The interval between admission date and screening date must be less than one. |
| Community Acquired Infection – Admission | CAI-A | Severity: 'Infected’  Classification: Not 'Hospital' or Healthcare acquired  The interval between admission date and screening date must be less than one. |

**Table A2.** Application of Model Comparison Algorithm in Manning and Mullahy 2001 [29]

| **Step** | **Result Cost** | **Result LOS** | **Recommendation** |
| --- | --- | --- | --- |
| Test kurtosis of log-scale residuals. If >3, consider semilog OLS model. | Kurtosis of 0.935 was found. | Kurtosis of 1.034 was found. | Not leptokurtic (kurtosis >3), GLM model should be used. |
| Apply modified Park test to select optimal GLM estimator. | Coefficient of 1.92. | Coefficient of 2.42. | Coefficient ~2, Gamma link function should be used. |

**Table A3.** Cost per MRSA Case, updated from Tübbicke et al 2012 [36]

| **Study, Timeframe, Location** | **Type of study** | **Outcome** | **2016 CAD** |
| --- | --- | --- | --- |
| Reed et al., July 1996 - Aug 2001, USA | Prospective cohort study | Full cost of hospitalization with MRSA infection at admission, US $28300 | $57,930 |
| Nelson, October 2007 - Sept 2010, USA | Method comparison: time dependent, conventional, matched | Incremental cost of MRSA infection, nosocomial US $24020 | $28,860 |
| Filice, Jan 2004 June 2006, USA | Comparison of MSSA to MRSA using semilog OLS | Total mean cost of MRSA infection, US $34660 | $50,200 |
| Cosgrove, July 1997 - June 2000, USA | Incremental cost of MRSA vs MSSA patients | Mean attributable cost of MRSA vs MSSA infection US $6920 | $14,460 |
| Ben David, Jan 2000 -Aug 2003, USA | Propensity score matched MRSA and MSSA infected patients | Total cost of MRSA infection, nosocomial, general unit US $53410 | $108,800 |
| Abramson and Sexton, Dec 1993- March 1995, USA | Pairwise-matched case–control study | Nosocomial MRSA infection incremental cost US $27080 | $55,750 |
| Murthy et al., 2006, Switzerland | Cohort study, decision analytic Markov model | Incremental cost of infection at admission or during stay US $8,290 | $8,890 |
| Gavalda et al., 2002, Spain | Cohort study | Incremental cost of infection, at admission or during stay €2730 | $5,150 |
| Chowers, 2005-2011, Israel | A single center, matched, historical cohort study and cost analysis | Net cost attributable to hospital acquired MRSA US $9400 | $11,300 |
| Wernitz et al., Apr 2001 -Nov 2002, Germany | Cohort study | Total cost for patients with infection, at admission or during €7770 | $14,180 |
| Resch et al., 2004, Germany | Retrospective matched-pair analysis | Incremental cost of infection and colonization vs matched, at admission or during €8200 | $16,260 |
| Herr et al., Aug 1999 - Aug 2000, Germany | Cohort study | Avoidable/ attributable cost due to MRSA colonization €9260 | $18,660 |
| Chaix et al., Jan 1993 - June 1997, France | Case–control study | MRSA attributable cost for ICU (nosocomial) MRSA infection US $9280 | $18,740 |
| Papia et al., June 1996- May 1997, Canada | Case–control study | Full cost of colonization, at admission or during stay $5240 | $7,460 |
| Kim et al., Apr 1996 - March 1998, Canada | Chart review, attributable cost of MRSA | Attributable inpatient cost of MRSA colonization of $1360 | $1,940 |
| Kim et al., Apr 1996 - March 1998, Canada | Chart review, attributable cost of MRSA | Attributable inpatient cost of MRSA infection, mean cost $14360 | $20,460 |
